# The interplay between host immunity and *Clostridioides difficile* infection

**DOI:** 10.1128/mbio.03562-24

**Published:** 2025-07-01

**Authors:** Danyang Li, Pedro H. V. Saavedra

**Affiliations:** 1 Department of Biology, Northeastern University 1848, Boston, Massachusetts, USA; The Ohio State University, Columbus, Ohio, USA

**Keywords:** *Clostridioides difficile*, host immunity, host-pathogen interactions, microbiota, infectious disease

## Abstract

*Clostridioides difficile* infection (CDI) is a major public health concern and the leading cause of healthcare-associated infectious enteric inflammation worldwide. Disruption of the gut microbiome predisposes to *C. difficile* colonization, proliferation, and production of cytotoxic toxins that damage the intestinal epithelial layer. CDI treatment is challenging in part due to the emergence of antibiotic-resistant strains and the lack of efficient vaccines, predisposing individuals to recurrent CDI episodes. Consequently, there is an urgent need for the development of novel therapeutic approaches. Both innate and adaptive immune responses contribute to protection against CDI, but the cellular and molecular mechanisms underlying this process are not completely understood. In this mini review, I discuss the history and recent findings with a focus on mechanisms that drive host immunity to *C. difficile*, with a conclusion on where the field stands and outstanding questions that remain elusive.

## INTRODUCTION

*Clostridioides difficile* is a gram-positive, toxin-producing, and spore-forming, anaerobic bacterium. *C. difficile* infection (CDI) is a major public health concern and one of the leading causes of healthcare-associated severe gastrointestinal infections worldwide, affecting over 500,000 people each year in the United States (1). CDI mainly occurs in vulnerable individuals such as patients on antibiotic treatment or other therapies that disrupt the gut microbiome (e.g., chemotherapeutic drugs), thereby promoting an optimal environment for *C. difficile* growth and intestinal colonization. The treatment for CDI presents significant clinical challenges, particularly due to the growing resistance of *C. difficile* against antibiotics used to treat CDI, such as vancomycin, fidaxomicin, and metronidazole, as well as broad-spectrum fluoroquinolone antibiotics that confer a colonization advantage in the antibiotic-disrupted intestinal microenvironment (2, 3). Moreover, recurrent *C. difficile* infection (rCDI) poses a significant challenge, with recurrence affecting up to 35% of initial CDI patients, and of those, up to 60% may suffer from further recurrences, resulting in multiple CDI episodes (4). In addition, the proportion of community-associated CDI cases has increased from 47% in 2012 to 53% in 2019 (5). Trehalose metabolism as a part of human diets has been suggested to contribute to the emergence of hypervirulent *C. difficile* strains. However, subsequent research indicated that the trehalose metabolic pathway is conserved among multiple lineages of *C. difficile* strains but not limited to the hypervirulent strains, suggesting additional mechanistic determinants may underlie the observed association between trehalose metabolism and increased clinical mortality in *C. difficile* infections (6, 7). Despite the availability of antibiotic therapies for CDI, mortality rates remained relatively stable between 2011 and 2017, with approximately 20% of patients experiencing disease recurrence likely in part due to the rise of antibiotic-resistant strains (8, 9). In addition to bacteria-related factors such as spore and biofilm formation, the frequent recurrence of CDI also suggests a failure to mount a protective, specific antibody response, potentially due to the disrupted gut microbiota that often precedes CDI (10–12). This is consistent with evidence demonstrating the crucial role of microbiota-derived metabolites in the development of T follicular helper (Tfh) cells, B-cell metabolism, and antibody production (13, 14). These observations highlight the critical need for alternative therapeutic strategies against CDI.

*C. difficile* spores are extremely resistant to oxygen, chemicals, and harsh conditions (15). Ingestion of spores is thought to be the main route of infection. In agreement, strains that are unable to form spores are not capable of persisting in such environments and spreading among hosts (15, 16). Importantly, sporulation is counteracted by host factors and commensal microbes. Spore germination to toxin-producing vegetative cells is a process that takes place in the small intestine in the presence of a specific primary bile acid germinant, namely, taurocholate. When nutrients become scarce, *C. difficile* cells cease growth and initiate sporulation. This well-described and regulated process is triggered by sensor histidine kinases, followed by phosphorylation and activation of the transcription factor Spo0A (17, 18). Notably, *C. difficile* spores were shown to be internalized by intestinal epithelial cells (IECs) and macrophages, retaining their viability and ability to germinate (10, 19).

CDI is primarily driven by two large pathogenic toxins, TcdA (308 kDa) and TcdB (270 kDa). While both contribute to CDI pathology in humans and animal models, TcdB is suggested to induce more severe effects (20–22). Although *C. difficile* also produces the ADP-ribosyltransferase binary toxin CDT, studies in mouse models of CDI utilizing CDT mutant strains have shown conflicting results, and further research is warranted to conclusively establish the physiological role of CDT during CDI (20, 23). TcdA and TcdB share a similar structure, comprising a C-terminal repetitive oligopeptides domain (CROPs), a cysteine protease domain, a transmembrane domain, and an N-terminal glucosyltransferase domain (GTD). Both TcdA and TcdB enter cells via receptor-mediated endocytosis, utilizing multiple receptors, potentially in a cell-type-specific manner (24). For instance, rabbit sucrase-isomaltase and gp96 have previously been shown to function as TcdA receptors (25, 26). Moreover, recent studies reported that sGAGs, LDLR, and LRP1 are all important cellular factors mediating the binding and entry of TcdA (27, 28). TcdB has been shown to bind to CSPG4, NECTIN3 (PVRL3), FZD1/2/7, and TFPI (29–33). Following endocytosis, the toxins undergo phagosome acidification-dependent autoprocessing, releasing the catalytic N-terminal fragment into the cytosol. The GTD of both toxins acts as a mono-glucosyltransferase that uniquely modifies a conserved threonine residue on Rho, Rac, and Cdc42 GTPases, disrupting their ability to bind GTP (34). Monoglucosylation-mediated inactivation of Rho GTPases leads to disruption of cytoskeleton dynamics and cellular integrity. While both toxins ultimately cause cell death, they have been suggested to mediate this process through distinct mechanisms. TcdA was proposed to induce apoptosis in a glucosyltransferase-dependent manner (35), whereas TcdB exhibits dose-dependent cytotoxicity through different mechanisms. At a low dose, TcdB induces glucosyltransferase-dependent apoptosis via Rho GTPase inactivation and caspase activation, similar to TcdA, while a higher dose of TcdB triggers glucosyltransferase-independent, NADPH oxidase-mediated reactive oxygen species (ROS) production, leading to oxidative stress and necrotic cell death (36). During CDI, IECs are the primary target of TcdA and TcdB. A comprehensive study showed that TcdA and TcdB mediate cell death via the intrinsic apoptosis pathway in IECs (37), resulting in tissue barrier breakdown, recruitment, and activation of immune cells in the colonic lamina propria. The clinical presentation of CDI varies widely among patients, with some individuals displaying asymptomatic colonization, whereas others develop symptomatic disease and high risk of recurrence (1). Unresolved CDI may thus result in intestinal inflammation that leads to a spectrum of symptoms ranging from mild diarrhea to life-threatening colitis that may involve severe ileus, toxic megacolon, colonic perforation with subsequent peritonitis, and in rare cases, septic shock (1). This illustrates the complex interactions between host immunity and *C. difficile*.

## INNATE IMMUNE RESPONSES TO *C. DIFFICILE*

**Fig 1:**
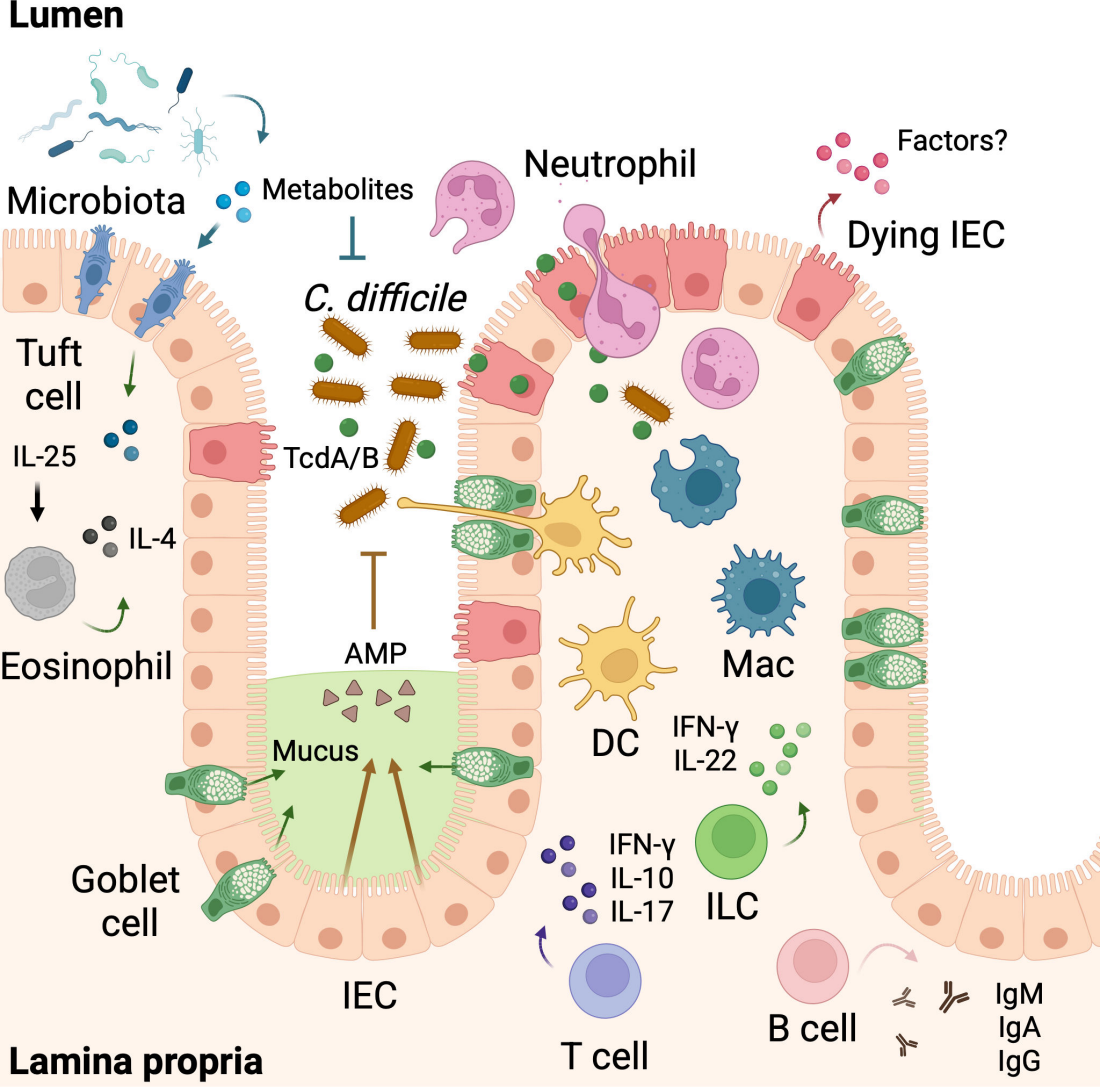
Host immune responses to *C. difficile* infection (CDI). The intestinal immune landscape in response to *C. difficile* infection involves a complex interplay between epithelial cells, immune cells, and microbiota-derived molecules. In the intestinal lumen, the gut microbiota competes for nutrient sources and produces metabolites that suppress *C. difficile* growth and colonization. Goblet cells and epithelial cells produce mucus and antimicrobial peptides (AMPs) that sustain intestinal homeostasis. Upon disruption of the microbiota, *C. difficile* is capable of proliferating and producing the cytotoxins (TcdA and TcdB) that induce intestinal epithelial cell (IEC) apoptosis. Damaged and dying IECs lose their ability to secrete mucus and AMPs but may release unknown factors that contribute to CDI protection. Concomitant to dysbiosis, accumulation of certain microbiota-derived metabolites such as succinate promotes expansion and activation of tuft cells. In the lamina propria, tuft cell-derived IL-25 promotes IL-4-producing eosinophil recruitment, expansion, and activation. Neutrophils infiltrate infected tissues, engaging in phagocytosis and ROS production. Innate lymphoid cells (ILCs) in the lamina propria rapidly respond to CDI by producing cytokines such as IFN-γ and IL-22 that contribute to CDI control. Macrophages (Mac) and dendritic cells (DCs) coordinate another layer of antibacterial defense mechanisms by promoting phagocytosis and capturing antigens for antigen presentation for initiation of adaptive immune responses. Activated different subsets of T cells produce cytokines such as IFN-γ, IL-10, and IL-17 to protect against CDI. Finally, a subset of T cells supports B-cell differentiation and activation for production of neutralizing antibodies (IgM, IgA, and IgG) and generation of memory B cells.

Immune responses to *C. difficile* are multifaceted, with many different cell types playing distinct roles (Fig. 1). The gut microbiota provides the first barrier against CDI, followed by a mucus layer that prevents access to IECs. Antibiotic-induced dysbiosis reduces microbiota diversity, thereby diminishing bacterium-derived signals that normally stimulate mucin synthesis in the intestinal tract. Furthermore, antibiotics directly induce metabolic stress in goblet cells, impairing their mucin-secretory capacity (38, 39). Notably, *C. difficile* negatively impacts mucin production by goblet cells as demonstrated by decreased expression of *MUC2* in human pluripotent stem cells infected with *C. difficile,* but whether this occurs *in vivo* is unknown (40). In the absence of competing commensal microbes, *C. difficile* colonizes the gut and proliferates, targeting primarily the intestinal epithelium layer during infection (41). TcdA and TcdB are both internalized and induce IEC apoptosis via caspase-3 and caspase-7 (35, 37, 42, 43). Importantly, gene-targeted mice with specific deletion of caspase-3 and caspase-7 in IECs were shown to be more susceptible during the early stages of CDI (37). Although the mechanisms mediating this process are not completely understood, this suggests IEC apoptotic cell death as a defense mechanism against CDI. In addition, glucose metabolism by IECs is increased during CDI, and inhibition of glycolysis attenuates disease severity and mortality (44), indicating that manipulation of IEC metabolism could be a potential strategy to mediate protective host responses during disease. Following IEC cell death and loss of epithelial barrier integrity, *C. difficile*-secreted toxins and commensal bacteria invade the colonic lamina propria and systemic circulation in certain cases, leading to activation of resident immune cells and further cell recruitment that amplify tissue inflammation (45, 46). Most notably, *C. difficile* triggers production of the chemokine CXCL1 or its human equivalent ortholog, interleukin (IL)-8, and neutrophil recruitment to the site of infection via MyD88 and Nod1 signaling (47–49). Mice with global deficiency of MyD88 or Nod1 display increased mortality during CDI. Importantly, a functional MyD88 in IECs alone is sufficient to protect mice against CDI, while MyD88 signaling in dendritic cells (DCs) and macrophages appears to be dispensable (50). In agreement with a role for MyD88 during CDI, the MyD88-dependent Toll-like receptors (TLRs) TLR2, TLR4, and TLR5 were reported to mediate immune responses through recognition of *C. difficile* cell wall components, surface layer proteins, and flagellin, respectively (51–53). Neutrophil recruitment to the colonic lamina propria during CDI has been shown to be crucial for host protection because neutrophil depletion exacerbates susceptibility to CDI (47, 48), possibly due to decreased ROS production and systemic spread of commensal microbes (47, 54). In support of these findings in animal models, neutropenic patients are reported to be at high risk of developing severe CDI (55). In contrast to IECs, myeloid cells such as macrophages and dendritic cells express high levels of the cytosolic receptor Pyrin (encoded by *Mefv*) (37, 56), which forms an inflammasome complex in response to RhoA inactivation mediated by TcdA and TcdB intoxication (57–59). Inflammasome activation drives processing and activation of caspase-1, leading to maturation and secretion of the pro-inflammatory cytokines IL-1β and IL-18 and induction of a lytic and inflammatory type of cell death, termed pyroptosis, that is dependent on GSDMD and NINJ1-mediated plasma membrane pore formation and rupture, respectively (60, 61). Inflammasomes play a crucial role in host defense against pathogens and have been extensively reviewed (62–64). In the context of CDI, the physiological impact of inflammasomes is controversial. ASC-deficient mice exhibited significantly increased susceptibility to oral infection with *C. difficile* vegetative cells, as demonstrated by enhanced mortality and weight loss in a standardized murine model. Another study reported that ASC deficiency conferred protection against *C. difficile*-secreted toxin TcdA- and TcdB-induced intestinal injury and inflammation in a toxin instillation model (65, 66), highlighting differences between infection and toxin challenging, akin to what is observed with gram-negative bacteria infection and lipopolysaccharide-induced septic shock mouse models. However, global ablation of Pyrin in an established mouse model of CDI was dispensable for disease progression and recovery (37), suggesting that inflammasomes are not the physiological primary host defense mechanism during CDI but could play a role in the rare cases of CDI-induced sepsis. Macrophages are crucial in promoting innate immune responses against pathogens, but their role during CDI, aside from Pyrin inflammasome activation, is not well understood. *C. difficile* is not an intracellular pathogen, but spores were reported to be internalized by macrophages via phagocytosis and stimulate secretion of pro-inflammatory factors (67). Studies aimed at colonic macrophage depletion prior to infection with *C. difficile* may thus offer insights and clarify their physiological role. Although DCs are innate immune cells, they are also essential for recognizing and presenting antigens to initiate adaptive immune responses against microbial infections. Intoxication of DCs with TcdA and TcdB promotes their maturation and activation via mitogen-activated protein kinase (MAPK)-mediated NF-κB signaling (68, 69). Interestingly, DCs readily phagocytose TcdB-intoxicated IECs, a process that promotes the migration and secretion of cytokines (70), suggesting DC migration to the draining lymph nodes for antigen presentation. However, because the impact of other functional roles and different subsets of DCs during CDI is unknown, the use of more specific tools to target DCs, such as DTR-transgenic mouse lines targeting *Xcr1*, *Clec9a*, and *Zbtb46* during CDI, is warranted (71–75). Eosinophils are best known to promote inflammatory responses during allergic reactions and protect against parasitic helminth infections. Notably, eosinophils have been reported to be important for intestinal homeostasis (76) and to promote host defense against enteric pathogens via release of EPX and eosinophil extracellular DNA traps (EETs), akin to neutrophil extracellular traps (77–79). However, only recently did their role during CDI start to emerge. This came from an observation that reduced numbers of eosinophils correlated with increased CDI-related mortality (80, 81). In support of these findings, expansion and activation of IL-4-producing eosinophils by IL-25 or inhibition of TLR2 were shown to be associated with disease protection by maintaining intestinal epithelial integrity (82, 83). Other granulocytes, such as mast cells and basophils, appear to play a minor role during CDI. While mast cells have been shown to be activated by TcdA and TcdB and to be involved in TcdA-mediated intestinal inflammation (84–86), basophils do not play a significant role in enteric bacterial infections, including CDI (87). Innate lymphoid cells (ILCs) are rare tissue-resident lymphocytes that, unlike B and T cells, do not express antigen-specific receptors and have emerged as a crucial component of the intestinal immune system, responding to diverse environmental cues to maintain gut homeostasis (88). Mice that have defective ILC development were reported to be highly susceptible to CDI (89, 90). Further studies showed that ILC1 played a dominant role with minor cooperation of ILC3 in protecting against *C. difficile* via induction of IL-22 and interferon gamma (IFN-γ)-mediated responses (90). In agreement, IL-22 was shown to prevent commensal microbe invasion following *C. difficile*-induced intestinal injury (45). Whether other cell types that are major IFN-γ producers, such as natural killer (NK) cells, also contribute to CDI protection remains to be elucidated. Because deletion of *Nfil3*, a gene involved in NK cell and ILC development, increases susceptibility to CDI (89), it will be important to evaluate how specific NK cell targeting impacts the course of CDI. More recently, ILC3-derived granulocyte-macrophage colony-stimulating factor (GM-CSF) was shown to promote host defense during early CDI through neutrophil-mediated responses (91). IL-33 upregulated during CDI was demonstrated to induce expansion and activation of ILC2 that mediated protection from infection via production of IL-5 and IL-13, which in turn would support eosinophil-mediated responses (92). Together, these studies show the importance of the innate immune system during CDI.

## ADAPTIVE IMMUNE RESPONSES TO *C. DIFFICILE*

The adaptive immune system promotes pathogen-specific cellular and antibody responses as well as the ability to generate memory cells for long-lasting protection. Increased frequency of rCDI indicates that the innate immune system is unable to provide broad protection against CDI beyond the acute phase of disease but also suggests that the adaptive immune system fails to establish a robust immune memory following CDI. Indeed, neutralizing immunoglobulins (Ig) such as IgM, IgG, and IgA targeting *C. difficile* toxins have been shown to be reduced in rCDI patients, whereas high Ig levels were detected in asymptomatic *C. difficile* carriers (93–95). Moreover, a patient with IgG deficiency suffering from multiple episodes of rCDI benefited from IgG infusions, highlighting its clinical relevance (96). Tfh cells support B-cell differentiation and activation for optimal antibody production and generation of memory B cells (97). Importantly, studies indicate that *C. difficile* infection induces a poor antibody response due to defective Tfh and memory B-cell expansion (98, 99), which may explain the high incidence of rCDI. In line with these findings, *C. difficile* TcdB was suggested to suppress antibody recall responses and germinal center formation by modulation of CXCR4-dependent B-cell migration, which could be rescued by a Food and Drug Administration (FDA)-approved drug targeting CXCR4 (100). In addition, IL-33 signaling in ILC2s has recently been shown to restore Tfh expansion and function following CDI (101). These studies thus offer new avenues for therapies aimed at manipulating antibody responses for vaccine development against CDI.

The cellular adaptive immune response mediated by T cells during CDI is also important for protecting the host. T helper 1 responses are associated with the expansion of IFN-γ-producing CD4^+^ T cells. However, CD4-deficient mice were not susceptible to infection with *C. difficile* (102), but defective IFN-γ is associated with worsened disease (90), indicating that IFN-γ produced in early stages of infection by ILCs, and possibly NK cells, plays a major role in host protection. Induction of Th17 responses coupled to the expansion of IL-17-producing CD4^+^ T cells is also observed in CDI. IL-17 production was shown to correlate with CDI resistance in young humans and mice (103). Moreover, another study found that increased levels of *C. difficile* TcdB-specific Th17 cells are linked to lower frequency of rCDI (104). Expansion of IL-17^+^CD4^+^ T cells is also associated with intestinal immunopathology during infection (105). Interestingly, Th17 cells generated following an acute mouse model of intestinal inflammation increased susceptibility to infection with *C. difficile* (106), suggesting that the context in which Th17 cells are generated, along with local signals, may influence their function in health and disease. Importantly, Th17 cells are elevated in inflammatory bowel disease (IBD) patients (105), which might explain why these patients are at increased risk of developing severe CDI (107). CD4^+^Foxp3^+^ T regulatory cells (Tregs) are crucial for suppressing autoimmunity and maintenance of homeostasis across tissues. Elevated numbers of Tregs are observed in rCDI patients (108). Moreover, genetic depletion of Tregs in *C. difficile*-infected mice leads to increased recruitment of neutrophils in the large intestine lamina propria (109). The majority of Tregs are of thymic origin, but a subset of Tregs has been reported to be generated extrathymically, including colonic Tregs (110, 111). While these few studies suggest that a balanced Treg response is important for controlling CDI, the relative contribution of thymic and extrathymic Tregs is unknown and warrants further investigation.

Other less studied cell types such as invariant NKT (iNKT) and mucosal-associated invariant T (MAIT) cells have also been implicated in CDI. iNKT cells are a subset of T cells with a restricted T-cell receptor (TCR) that recognizes glycolipid antigens presented by CD1d-bearing antigen-presenting cells (112). In this context, iNKT cells have been shown to contribute to Tfh-mediated antibody responses against *C. difficile* polysaccharide II and TcdB (113, 114). In addition to mediating colonization resistance, the gut microbiota also affects CDI by shaping iNKT cell functions, including immunoglobulin class switching by providing diverse antigens through CD1d presentation and releasing PAMPs, while iNKT cells, in turn, modulate microbial composition via cytokine secretion, which could potentially affect the occurrence of CDI (115, 116). Similar to iNKTs, MAIT cells harbor a semi-invariant TCR that recognizes microbial vitamin B metabolites presented on major histocompatibility complex class-I related protein (MR1) (117). In addition, MAIT cell development is dependent on the host microbiota (118). Studies showed that *C. difficile* infection in mice and humans induces a robust inflammatory response in MAIT cells (119, 120). Despite their activation during CDI, genetic ablation of MAIT cells resulted in increased resistance to *C. difficile* colonization in mice, which was shown to be dependent on the microbiota present in MAIT cell-deficient animals (121), suggesting that MAIT cells modulate the microbiota composition. Nevertheless, adaptive immune cells are a critical host component that mediates protection against *C. difficile*.

## MICROBIOTA-MEDIATED RESPONSES TO *C. DIFFICILE*

The host microbiome is a crucial line of defense against CDI. The prime example is illustrated by the fact that CDI occurs mainly when the microbiota balance is disrupted (122). Several factors contribute to gut dysbiosis, including widespread use of broad-spectrum antibiotics, diet, chemotherapy, and genetics (123). Therefore, therapy strategies aimed at diversifying the gut microbiota, such as fecal microbiota transplant (FMT), probiotics, and dietary modulation of the microbiome, are more likely to be successful in disease prevention and treatment, given the emergence of antibiotic-resistant strains (124). Perhaps the best evidence to highlight how diversity in the microbiota is beneficial in preventing CDI was a small clinical trial demonstrating the efficacy of FMT for primary CDI (125). Further studies showed that FMT was also effective in resolving recurrent cases of CDI (126, 127). Notably, the FDA has recently approved two fecal microbiota products to prevent rCDI (128, 129), underscoring its powerful benefit. Importantly, the efficacy of FMT against CDI appears to be dependent on CD4^+^Foxp3^+^ Treg cells (109).

Commensal microbes produce a myriad of metabolites that regulate host physiology across tissues and shape the gut microbiome diversity. Bile acids are molecules essential for host metabolism derived from dietary fat and synthesized in the liver. Primary bile acids are conjugated to taurine or glycine and released into the small intestine in response to food to support digestion. The majority of primary bile acids are reabsorbed in the gut and circulate back to the liver, but a small pool reaches the large intestine, where specific members of the microbiota convert primary bile acids into secondary bile acids that possess diverse cellular functions (130). While primary bile acids such as taurocholic acid promote *C. difficile* growth and spore germination, gut microbiota-derived secondary bile acids that include lithocholic acid and ursodeoxycholic acid inhibit *C. difficile* spore germination and proliferation (131, 132). Importantly, a study identified *Clostridium scindens* as a secondary bile acid-producing strain that could be used therapeutically to mediate resistance against CDI (133). Surprisingly, a study suggested that while a microbiome enriched in secondary bile acid-producing strains correlates with an environment that promotes resistance to *C. difficile* growth, this protection was independent of bile acids (134). Intra- or interspecies microbe-microbe interactions can also impact *C. difficile* colonization capacity. For instance, naturally occurring avirulent *C. difficile* strains have been shown to promote protection against infections with virulent *C. difficile* strains (135–138). The gut commensal *Clostridium butyricum* was shown to prevent *C. difficile* colonization by reducing the levels of intestinal succinate, a short-chain fatty acid (SCFA) derived from microbiota breakdown of dietary fiber and used as an energy source for *C. difficile* proliferation (139). In addition, decreased succinate levels correlated with diminished production of the pro-inflammatory cytokine tumor necrosis factor alpha (TNF-α) and augmented protective T cell-mediated responses, thereby promoting gut epithelial integrity (139). Interestingly, a commensal protozoan was shown to protect against CDI by increasing intestinal IFN-γ and modulation of arginine-ornithine metabolism, affecting host immunity and *C. difficile* virulence (140, 141). Dysbiosis may also promote *C. difficile* growth by expansion of antibiotic-resistant opportunistic pathogens that produce chemicals leveraged by *C. difficile*, such as enterococci, which are reported to be present in patients infected with *C. difficile* (142). In agreement, a study showed that *Enterococcus* produces several amino acids that enhance *C. difficile* colonization and pathogenesis through *C. difficile* metabolic reprogramming (143). Thus, other members of the gut microbiome that modulate arginine metabolism could potentially play a role in enhancing *C. difficile* fitness and pathogenesis (144–146).

Nutritional modulation of the microbiome has become increasingly recognized as an important factor accounting for susceptibility to *C. difficile* infections. A diet rich in fiber has been linked with a diverse microbiome and protective immune responses against CDI (147). Most notably, SCFAs derived from microbiota-mediated breakdown of dietary fiber, such as butyrate, acetate, succinate, and valerate, are important modulators of intestinal immunity and have been shown to impact *C. difficile* growth (148). Butyrate has been shown to suppress inflammatory responses in the large intestine (149). Interestingly, butyrate prevents CDI by directly inhibiting its growth or by modulation of colonic epithelial cell resistance to *C. difficile* toxins (150, 151). Accordingly, colonization with butyrate-producing commensals protects the host against CDI (152, 153). Acetate mediates protective responses against *C. difficile* by promoting neutrophil recruitment, enhancing production of IL-22 by ILC3s, and reducing expansion of pathogenic CD4^+^ intraepithelial lymphocytes through downregulation of MHC-II expression on IECs (154, 155). Valerate levels were shown to be decreased in CDI patients and significantly increased after FMT, which was accompanied by a drastic reduction in *C. difficile* numbers. Moreover, valerate also prevents *C. difficile* growth without compromising other commensal bacteria (156). In contrast to butyrate and acetate, succinate levels are elevated following antibiotic treatment and favor *C. difficile* proliferation and also promote inflammatory responses (157). In agreement with succinate in supporting *C. difficile* proliferation, a *C. difficile* mutant unable to metabolize succinate fails to colonize the host and establish an infection (158). Thus, the development of molecules aimed at targeting *C. difficile* metabolic pathways may pave the way for a new class of therapies. Opposed to its role directly on *C. difficile*, succinate-mediated expansion of tuft cells in the colon was recently shown to protect against CDI (159), most likely as a way to offset expansion of microbes that facilitate pathogen colonization. These data illustrate how succinate can have opposing effects in a context-dependent manner.

In contrast to dietary fiber, several other types of enriched diets have been linked with increased susceptibility to CDI. For instance, a high-fat diet decreases the abundance of *Lachnospiraceae* and *Ruminococcaceae*, which normally compete for nutrients, transform primary into secondary bile acids, and prevent *C. difficile* growth (160, 161). A diet rich in protein promotes the expansion of *Lactobacillus* that, in turn, increases the generation of amino acids through fermentation that are metabolized by *C. difficile* to support its growth (160, 162, 163). Similarly, a diet enriched in zinc was shown to decrease the microbiota diversity accompanied by expansion of *Enterococcus* and elevated levels of the zinc-binding protein calprotectin (164). Although high levels of calprotectin are associated with severe CDI (165, 166), calprotectin deficiency increases susceptibility to CDI due to failure in limiting organismal zinc levels (164). Overall, these studies illustrate the diverse mechanisms by which the microbiota impacts *C. difficile* colonization and infection establishment.

## IMMUNE-BASED THERAPEUTIC STRATEGIES

The standard line of treatment against CDI is the antibiotics fidaxomicin, metronidazole, and vancomycin. However, resistance of some *C. difficile* strains against these antibiotics is a major public health concern, underscoring the need for alternative therapeutic and preventive approaches. Here, we will briefly focus on reviewing the history of available immunological agents and discuss novel developments in immune-based therapies against CDI. Because IgG levels against *C. difficile* toxins are elevated in the serum of patients with asymptomatic colonization and CDI is suggested to impair the generation of antibody responses, vaccine development and antibody treatment against *C. difficile* toxins are an attractive approach for CDI prevention and treatment. Although administration of IgG against *C. difficile* toxins has been reported to aid in the resolution of CDI (167), no clinical trials were performed for robust evaluation. Nevertheless, these results suggested that specific monoclonal antibodies could serve as a potential treatment for CDI. Consistent with this finding, two monoclonal antibodies targeting *C. difficile*, TcdA (actoxumab) and TcdB (bezlotoxumab), were identified and tested in clinical trials (168). While bezlotoxumab reduced the rate of rCDI, the addition of actoxumab did not improve treatment efficacy, suggesting that neutralization of TcdB may be more beneficial than TcdA targeting (169). Importantly, bezlotoxumab received FDA approval for rCDI treatment in 2016 and became available for use in 2017 but was discontinued at the end of January 2025. Another TcdB monoclonal antibody, AZD5184 (previously known as PA41), is undergoing phase I clinical trials (170). Because access to antibody therapy may not be widely available, vaccine development targeted against *C. difficile* toxins and other proteins remains clinically relevant and is the focus of ongoing research. Vaccines against *C. difficile* flagellin, spore, and vegetative cell surface proteins would in theory have the potential to target and eliminate *C. difficile* at early stages before an infection could be established. Notably, while these vaccines have been shown to be immunogenic, only a flagellin-based vaccine protects against *C. difficile* colonization and infection in mice (171–173), but further studies and trials in humans are necessary to evaluate its efficacy. On the other hand, several vaccines targeting *C. difficile* TcdA and TcdB were evaluated in clinical trials (174). Sanofi (NCT01887912) and Pfizer (NCT03090191) developed promising vaccines based on detoxified TcdA and TcdB, but they failed to prevent primary infection, resulting in termination of their phase III clinical trials (173). A vaccine based on recombinant chimeric fusion protein of the CROP domains of both TcdA and TcdB was developed by Valneva (175), which has finalized its phase II clinical trial (NCT02316470). Finally, an oral vaccine based on a live non-toxigenic *C. difficile* strain was developed by Shire (now Takeda) (176) and also completed a phase II clinical trial (NCT01259726). Valneva vaccine candidate VLA84 is on hold, but the company expressed interest in reactivating the program to move into phase III clinical trial. The Shire vaccine candidate NTCD-M3 (former VP20621) was expected to initiate a phase III clinical trial in 2024, in partnership with Destiny Pharma and Sebela Pharmaceuticals.

The emergence of messenger RNA (mRNA) vaccines following the COVID-19 pandemic opened up a powerful tool for vaccine development against a variety of infectious pathogens (177–180). In a recent study, it was shown that nucleoside-modified mRNA, namely, lipid nanoparticle-mRNA (LNP-mRNA) multivalent vaccines targeting multiple *C. difficile* virulence factors and spore proteins, induced a robust antigen-specific mucosal antibody response, expansion of Tfh cells and germinal center B cells (181). LNP-mRNA vaccines protected mice against intoxication with isolated *C. difficile* TcdA and TcdB as well as primary CDI. In contrast to other immunization strategies, LNP-mRNA vaccines induced long-lasting immunity and protected against rCDI, suggesting efficient memory B-cell generation. Importantly, LNP-mRNA vaccines did not impact the composition of the gut microbiota, demonstrating its suitability against CDI. Fascinatingly, LNP-mRNA vaccination also induced robust immune responses in aged non-human primates (181). This study thus reinforces the potential of mRNA vaccines and provides a blueprint for the development and introduction of mRNA-based vaccines against CDI in humans.

## OUTSTANDING QUESTIONS AND CONCLUDING REMARKS

In this mini review, we sought to provide a comprehensible background and focus on novel findings relating to the interplay between host immunity and CDI (Fig. 1). Undoubtedly, CDI remains a significant public health concern and poses a high burden on a growing elderly society due to high recurrence rates as a result of the emergence of hypervirulent strains. Disruption of a diverse and healthy microbiota often precedes CDI and contributes to compromised immune responses and impaired development of a protective antibody response. In addition, *C. difficile* strains resistant to conventional and other narrow-spectrum antibiotic treatment underscore the urgent need for novel therapeutic strategies to effectively treat CDI. While many advances and discoveries were made over the past few years, several questions remain to be elucidated. For instance, neutrophils have been shown to play both protective and detrimental responses during CDI. However, the cellular and effector mechanisms leading to these differential responses are not completely understood. Neutrophils were shown to be closely localized to damaged IECs (47). At the same time, IEC apoptosis was shown to provide host protection during early stages of CDI, but the mechanisms behind this process are unknown (37). It is tempting to speculate whether damaged IECs activate pathways downstream of apoptosis that release signals to instruct innate immune responses early during infection. In addition, phagocytosis of dying cells—termed efferocytosis—is a process mediated by professional and non-professional phagocytes that promote wound healing and are partially dependent on the microbiome-derived SCFA butyrate (182–184), but whether efferocytosis also contributes to epithelial layer regeneration following CDI-induced intestinal injury is unknown. Furthermore, the cellular interaction network between immune and non-immune cells in CDI is a vast resource to be explored, which, coupled with new technologies such as spatial transcriptomics and single-cell RNA sequencing, could offer new insights into molecular pathways that could be targeted to manipulate host immunity for protection against CDI. In conclusion, these and many other challenges remain to fully understand the pathogenesis of CDI, but recent advances are paving the way to close this knowledge gap.
